# Reversible suppression of hypothalamo–pituitary–adrenal axis in Addison’s disease due to ethinyl oestradiol-induced increase in total cortisol

**DOI:** 10.1530/EDM-24-0055

**Published:** 2024-12-19

**Authors:** Krzysztof C Lewandowski, Monika Głuchowska, Małgorzata Karbownik-Lewińska, Andrzej Lewiński

**Affiliations:** ^1^ Department of Endocrinology and Metabolic Diseases, Medical University of Lodz, Lodz, Poland; ^2^ Faculty of Medicine, Mazowian University of Plock, Plock, Poland; ^3^ Department of Pathology of Pregnancy, 1st Chair of Gynaecology and Obstetrics, Medical University of Lodz, Lodz, Poland; ^4^ Department of Endocrinology and Metabolic Diseases, Polish Mother’s Memorial Hospital – Research Institute, Lodz, Poland

**Keywords:** Addison’s disease, hypothalamic–pituitary–adrenal axis, ethinyl oestradiol, cortisol

## Abstract

**Summary:**

An oral contraceptive pill (OCP)-induced increase in total cortisol lead to reversible suppression of the hypothalamic–pituitary–adrenal (HPA) axis and insulin resistance (IR) in a patient with Addison’s disease. We suggest that this might influence the choice of an OCP in such patients. A 20-year-old female was diagnosed with Addison’s disease (cortisol: 44 nmol/L, adrenocorticotropic hormone (ACTH): >500 pg/mL) and started on hydrocortisone (HC). Few months later, an OCP (30 μg ethinyl oestradiol (EE) and 3 mg drospirenone) was added. Total cortisol was above the upper assay detection limit (UADL), while ACTH was inappropriately ‘normal’: cortisol 8:00 (pre-dose) 83 nmol/L, post-dose 10:00 >1757 nmol/L, ACTH 8:00 (pre-dose) 24.1 pg/mL and post-dose 10:00 3.8 pg/mL. Even 5 mg of oral HC induced an increase in cortisol above UADL. The glucagon stimulation test (GST) showed brisk growth hormone secretion. The corticotropin-releasing hormone (CRH) test showed partial hypothalamic suppression of CRH release: minimal ACTH 42.4 pg/mL and maximal ACTH 87.3 pg/mL, i.e. relatively low levels for all cortisol concentrations <69 nmol/L. Withdrawal of the OCP resulted in the return of high ACTH concentrations typical for patients with Addison’s disease on HC replacement. There was also a marked improvement in insulin resistance (a fall in homeostasis model assessment - insulin resistance (HOMA-IR) from 3.64 to 1.69 and a marked decline in mean insulin concentrations during GST). EE administration resulted in a massive increase in total cortisol with suppression of the HPA axis and IR suggestive of relative hypercortisolaemia. This raises the question of whether EE should be avoided as a contraceptive agent in women with adrenal failure.

**Learning points:**

- An OCP containing 30 μg EE induced relative and reversible hypercortisolaemia in a patient with Addison’s disease with evidence of suppression of ACTH secretion on dynamic pituitary function tests.
- We suggest that, in some patients with adrenal failure, EE administration may lead to unrecognised relative hypercortisolaemia and IR.
- There is literature evidence that, in patients with Addison’s disease, EE may decrease cortisol clearance.
- These alterations are reversible upon EE withdrawal and may have implications for the choice of a contraceptive agent in women with Addison’s disease.

## Background

Adequate adjustment of hydrocortisone (HC) dose in patients with adrenal deficiency is not straightforward, and several methods of dose adjustments have been proposed (1). In patients not taking an oral contraceptive pill (OCP), HC (cortisol) is bound to both cortisol-binding globulin (CBG) and albumin, while CBG is typically saturated for concentrations above 550 nmol/L (2). This is, however, not true for oral contraceptive users, as oestrogenic components of an OCP increase CBG and thus total cortisol concentrations (3, 4). Ditchell and coworkers (5) strongly recommend that total cortisol should not be used to assess the adrenal axis in oral contraceptive users. We confirm these observations and present a case of relative and reversible hypercortisolaemia with suppression of the hypothalamic–pituitary–adrenal (HPA) axis in a young woman with Addison’s disease that was induced by an OCP.

In our opinion, the existence of such a phenomenon is both under-investigated and most likely unrecognised by managing physicians; hence, our observation may have broader implications in terms of the choice of an oral contraceptive for women with Addison’s disease.

## Case presentation

A 20-year-old female with an autoimmune thyroid disease (anti-TPO antibodies 426 IU/mL, reference range <34) was diagnosed with Addison’s disease about 11 months prior to presentation in our department (cortisol: 1.59 μg/dL (44 nmol/L), ACTH: >500 pg/mL). She was started on HC 15 mg around 7:00–8:00 h and 10 mg around 13:00–14:00 h. No fludrocortisone was prescribed. Few months later, an OCP containing 3 mg drospirenone and 0.030 mg ethinyl oestradiol (EE) (Yasmin®) was added. She complained, however, of general malaise and a non-specified ill feeling, so further tests were performed. These showed a normal full blood count, normal liver function tests, glucose 4 mmol/L, TSH 1.41 μIU/mL (0.27–4.2) (without levothyroxine), 25OH-vitamin D 30.4 ng/mL and very high random cortisol above 60 μg/dL (1660 nmol/L). She was referred for further assessment in our department.

## Investigation

We subsequently assessed cortisol and ACTH concentrations after oral administration of 10 mg HC. Early morning cortisol was low with an inappropriately ‘normal’ ACTH, while all subsequent cortisol concentrations were above the upper assay detection limit (UADL), that is >63.44 μg/dL (>1757 nmol/L) (Table 1). ACTH concentrations oscillated around or below the lower reference range (Table 1). Extremely high total cortisol concentrations, i.e. above UADL, were also observed after oral administration of just 5 mg HC (Table 1). A similar situation was observed on the cortisol day curve (Table 2). In view of low ACTH concentrations, we subsequently assessed pituitary function during a 1.0 mg intramuscular glucagon stimulation test (GST). We also measured glucose and insulin concentrations during GST (Table 3) and calculated the homeostasis model assessment - insulin resistance (HOMA-IR) index according to the following formula: glucose (mmol/L) × insulin (μU/mL)/22.5 (6).

**Table 1:** ACTH and cortisol concentrations after oral administration of 10 or 5 mg HC at 8:00 in a 20-year-old female patient with Addison’s disease taking 30 μg EE and 3 mg drospirenone.

	HC dose	0 min*	30 min	60 min	90 min	120 min	150 min	180 min
ACTH (pg/mL)	10 mg	15.6	12.1	8.2	5.8	4.0	3.7	3.4
Cortisol	10 mg							
μg/dL		1.9	All values > 63.44					
nmol/L		52	All values > 1757					
Cortisol	5 mg							
μg/dL		1.7	All values > 63.44					
nmol/L		47	All values > 1757					

EE, ethinyl oestradiol; HC, hydrocortisone.

* pre-dose.

**Table 2:** Cortisol and ACTH concentrations during administration of 10 mg HC at 8:00 and 10 mg at 14:00 in a 20-year-old female patient with Addison’s disease before and after OCP withdrawal.

	8:00	10:00	14:00	16:00	18:00	HC dose	
						8:00	14:00
With OCP*							
Cortisol						10 mg	10 mg
μg/dL	3.0	>63.44	40.7	>63.44	–		
nmol/L	83	>1757	1127	>1757			
ACTH (pg/mL)	24.1	3.8	2.6	3.1	–	10 mg	10 mg
Without OCP							
Cortisol						10 mg	10 mg
μg/dL	<0.054	7.3	2.5	19.9	2.9		
nmol/L	<0.15	202	69	551	80		
ACTH						10 mg	10 mg
pg/mL	757	181.6	142.2	13.7	21.4		
% increase	3041	4678	5588	341	728		

HC, hydrocortisone; OCP, oral contraceptive pill.

* EE 30 μg + 3 mg drospirenone; ^†^pre-dose.

**Table 3:** Glucose, insulin, ACTH and cortisol concentrations during glucagon stimulation test (morning hydrocortisone dose omitted) in a 20-year-old female patient with Addison’s disease before and after OCP withdrawal.

	0 min	30 min	60 min	90 min	120 min	150 min	180 min
With OCP							
Glucose, mmol/L	3.83	7.06	6.78	5.94	80	4.44	4.06
Insulin, μU/mL	21.4	170.8	126.5	54.2	18.5	11.8	13.1
ACTH, pg/mL	19.4	16.1	12.5	13.2	20.2	18.9	20.2
GH*, ng/mL	1.76	42.88	26.53	10.41	3.59	3.96	13.1
Cortisol							
μg/dL	2.4	2.0	1.9	1.6	1.6	1.4	1.3
nmol/L	66	55	52	44	44	39	36
Without OCP							
Glucose, mmol/L	4.44	7.83	7.11	6.0	3.78	3.0	3.11
Insulin, μU/mL	8.57	70.5	49.1	27.2	7.1	5.2	3.0
% decline	59.6	58.7	61.2	49.8	61.6	55.9	77.1
ACTH, pg/mL	924	634	549	519	550	721	1057
% increase	4662	3837	4292	3831	2622	3714	5132
Cortisol							
μg/dL	All values < 0.054						
nmol/L	All values < 0.15						

EE, ethinyl oestradiol; GH, growth hormone; GST, glucagon stimulation test; OCP, oral contraceptive pill.

* Due to fully retained growth hormone secretion, we did not repeat growth hormone measurements during GST on OCP withdrawal; ^†^EE 30 μg + 3 mg drospirenone.

GST revealed low cortisol concentrations, inappropriately ‘normal’ ACTH without further increase after glucagon, and a brisk increase in growth hormone (Table 3).

A corticotropin-releasing hormone (CRH) test was also performed (Table 4) and showed an increase in ACTH from 42.4 to 87.3 pg/mL at 30 min post-CRH, however, without any increase in total cortisol (Table 4).

**Table 4:** CRH stimulation test in a 20-year-old female patient with Addison’s disease taking an OCP containing EE 30 μg + 3 mg drospirenone (morning hydrocortisone dose omitted).

	−15 min	0 min	15 min	30 min	60 min	90 min
Cortisol						
μg/dL	2.5	2.4	2.5	2.2	1.9	1.7
nmol/L	69	66	69	61	52	47
ACTH, pg/mL	42.4	44.5	63.3	87.3	76.4	58.0

EE, ethinyl oestradiol; GH, growth hormone; OCP, oral contraceptive pill.

## Treatment

The patient was subsequently asked to stop the OCP, while fludrocortisone (50 μg once a day) was added. She had been discharged on HC 10 mg twice daily and was admitted for re-evaluation three months later.

Her results during the subsequent hospital stay are presented in Tables 2 and 3. There was a marked increase in ACTH (several-fold above the upper reference limit), with pre-dose ACTH concentrations within the range observed in patients with Addison’s disease. Both fasting and glucagon-stimulated insulin concentrations were much lower following discontinuation of the OCP, as demonstrated by a fall in HOMA-IR from 3.64 to 1.69 and over a 50% reduction in insulin concentrations during GST (Table 3). Despite the addition of fludrocortisone, her renin concentrations increased from 40.06 to 110.70 µIU/mL, with an aldosterone concentration of 19.33 pg/mL, without the OCP.

## Outcome and follow-up

She had been followed up in the endocrine clinic for over a year without further complications.

## Discussion

The classical teaching implies that women taking a combined OCP have an increase in total cortisol without a significant change in free cortisol concentrations (7). This teaching has recently been confirmed in a trial comparing the effects of oestradiol- and EE-based contraceptives on adrenal steroids (8), where EE induced a mean increase in CBG from 25.42 to 67.33 μg/dL, however, without a significant change in the free cortisol index.

Nevertheless, clinical experience from Cushing’s syndrome has clearly demonstrated that, although single cortisol concentrations (particularly in the morning) may not be different for healthy subjects, yet, what matters is not a single concentration but an overall, i.e. 24-hour, exposure to cortisol excess (9).

In our patient with Addison’s disease, we demonstrated suppression of the hypothalamo–pituitary ACTH drive that was normalised on OCP withdrawal. Our case is different from a situation described previously by Lewandowski and coworkers (10), where, in long-standing Addison’s disease in a female patient not taking an OCP, there was suppression of ACTH secretion with CRH resistance. In that case, however, there was a fully retained ACTH response to hypoglycaemia during an insulin tolerance test, thus indicating possible exhaustion of the central CRH–ACTH drive most likely due to chronic overstimulation.

Regretfully, in our patient, we were unable to measure CBG levels, but a massive increase in total cortisol above UADL after oral administration of just 5 mg of HC (Table 1) might indicate that there was possibly a very striking increase in CBG concentrations. The average increase in CBG in patients taking 30 μg EE in the study by Kangasniemi and coworkers (8) was about 40 μg/dL, but the upper standard deviation limit oscillated about 70 μg/mL. Westhoff and coworkers (11) described CBG increments reaching 100 μg/dL in OCP-compliant patients taking a combination of EE and levonorgestrel.

This raises the question of whether an OCP-induced increase in CBG might modulate cortisol and ACTH metabolism. Kumsta and coworkers (12) assessed cortisol and ACTH responses during a controlled stress situation (the so-called Trier social stress test (TSST)). They reported that, in women taking oral contraceptives, CBG levels were negatively associated with ACTH and salivary cortisol and positively associated with total cortisol following TSST. They concluded that ‘CBG levels have to be taken into account as a potential modifier of ACTH and cortisol responses to psychosocial and pharmacological stimulation’. More detailed studies on cortisol metabolism in OCP users were performed by a group from a leading UK endocrine centre (13), where the authors assessed total and free serum cortisol pharmacokinetics following IV and oral HC administration in healthy volunteers (*n* = 6), oestrogen-treated women (*n* = 6) and patients with a CBG variant with no steroid-binding activity (CBG G237V homozygotes, *n* = 2), previously described by the same group (14). Although serum free cortisol concentrations were only slightly (and non-significantly) higher in OCP users, there was a marked increase in both total and free cortisol elimination half-life (e.g. mean 96 min for OCP users versus 61 min and 56 or 58 min for healthy volunteers and G237V homozygotes, respectively), accompanied by about a 50% reduction in both total and free cortisol clearance. These findings were confirmed by marked changes in salivary cortisol pharmacokinetics, as reflected by a marked increase in the concentration–time area under the curve and half-life both for salivary cortisol and for salivary cortisone, which is the product of free cortisol oxidation by 11-β-hydroxysteroid dehydrogenase type 2 in the parotid glands. The authors predicted that these increases in free cortisol half-life and clearance might be not important in patients with an intact HPA axis but stated that ‘in patients dependent on hydrocortisone replacement therapy, there may be a risk of overtreatment and isolated basal cortisol measurement is not sufficiently precise to identify these differences*’*.

In our opinion, we have just identified a case that fulfils the above prediction of possible cortisol overtreatment in patients with Addison’s disease taking an OCP. So far, this issue has remained completely under-recognised. Our patient demonstrated surprisingly ‘normal’ ACTH concentrations in the setting of a very low (pre-dose) total cortisol. There was also a very poor ACTH secretory response to glucagon with a retained response to CRH (thus indicating relative CRH suppression). Furthermore, there was also a marked improvement of HOMA-IR (1.69 vs 3.64) accompanied by an over 50% fall in insulin concentrations during GST, indicating an OCP-related induction of insulin resistance (IR), most likely related to alterations in cortisol metabolism. All these alterations indicate that hypothalamic sensors during EE administration developed adaptive changes in a fashion similar to glucocorticoid excess states. In our opinion, this hypothesis is substantiated by full restoration of ACTH secretion and an improvement in IR upon OCP withdrawal. Furthermore, renin concentration was also inappropriately low for her aldosterone level (most likely as a result of a mineralocorticoid activity of cortisol), while after discontinuation of the OCP, renin concentrations increased almost three-fold despite the addition of fludrocortisone. It should be noted that long-term cortisol over-replacement might be associated with glucose intolerance, hypertension and osteoporosis (15, 16).

Although our data are based on a single case, in our opinion, our observations may have important implications for numerous young women with Addison’s disease who choose to take oral contraception. So far, we cannot say how many women with Addison’s disease could be affected by such a relative OCP-induced cortisol excess. There is a question whether we should check morning (pre-dose) ACTH in women with Addison’s disease who take oral contraceptives in order to identify those possibly exposed to an excess of glucocorticoids. We note, however, a study by Thompson and coworkers (17), where the authors investigated total cortisol responses to either intravenous or oral administration of 20 mg HC in 27 patients with adrenal failure, including nine patients with Addison’s disease (two of them on an unspecified preparation of oral oestrogen, but only one patient of reproductive age (35 years), hence most likely taking an OCP). In that study, there was at least one case of exceptionally high concentrations of total cortisol, reaching concentrations close to 1600 nmol/L after oral and around 4500 nmol/L after intravenous HC administration, i.e. concentrations around 50% higher than all the remaining study subjects and close to cortisol concentrations observed in our case. We speculate that it is likely that those very high cortisol concentrations were observed in that 35-year-old female patient with Addison’s disease, possibly on an OCP.

### Summary and implications

In our opinion, our observations raise questions about the choice of oral contraceptive agents for women with Addison’s disease. EE is particularly potent in inducing an increase in total cortisol and CBG (18). Hence, at least in some patients with adrenal failure, EE administration may lead to unrecognised relative and reversible hypercortisolaemia and IR, likely due to an EE-induced increase in total cortisol and a decrease in cortisol clearance. On the other hand, there are data that contraceptives containing oestradiol valerate induce only minor alterations in total cortisol and CBG concentrations (8). Increases in total cortisol and CBG are also less pronounced in compounds containing 17β-oestradiol (18) or oestetrol/drospirenone versus EE/drospirenone combination (19). This raises the question whether EE-containing OCPs should be avoided in women with Addison’s disease. This issue, however, needs to be addressed in a separate study.

## Declaration of interest

The authors declare that there is no conflict of interest that could be perceived as prejudicing the impartiality of the work reported.

## Funding

This study was funded by statutory funds from the Medical University of Lodz (503/1-107-03/503-11-001), Lodz, Poland.

## Patient consent

Written informed consent was obtained from the patient for publication of the submitted article and accompanying images.

## Author contribution statement

KCL – clinical management of the patient in the department, conceptualisation and design, investigation, methodology, analysis and interpretation of data, writing of the manuscript. MG – management of the patient in the outpatient clinic, investigation and contribution to writing of the manuscript. AL – investigation, analysis of data, revision of content, and final approval of the version published. All authors contributed to the article and approved the submitted version.
